# The Relation between Volume and Outcome of Transcatheter and Surgical Aortic Valve Replacement: A Systematic Review and Meta-Analysis

**DOI:** 10.1155/2020/2601340

**Published:** 2020-04-18

**Authors:** Jialing He, Zhen Zhang, Han Wang, Lin Cai

**Affiliations:** Department of Cardiology, The Third People's Hospital of Chengdu, The Affiliated Hospital of Southwest Jiaotong University, China

## Abstract

Transcatheter aortic valve replacement (TAVR) and surgical aortic valve replacement (SAVR) are standard procedures for dealing with severe aortic stenosis patients. Researchers have not carried out a systematic review of the volume-outcome relationship in TAVR and SAVR. Our study is intended to address this problem. We systemically searched databases through MEDLINE, EMBASE, PUBMED, and the Cochrane Library up to September 2019. Two reviewers independently screened for the studies and evaluated bias. We used short-term mortality (in-hospital or 30-day mortality) as an outcome. A meta-analysis of TAVR with 115,596 patients ranging from 2005 to 2016 showed a result significantly in favor of high-volume hospitals (OR 0.43 (CI 0.36-0.51)). The subgroup of population period, region, data type, and cut-off value did not show any difference. A meta-analysis of SAVR comprising 418,384 patients ranging from 1994 to 2011 revealed that the OR of short-term mortality for a high-volume hospital compared with that of a low-volume hospital was 0.73 (CI 0.71, 0.74). No difference was observed in subgroups based on population period and cut-off. In conclusion, we found that short-term mortality was lower in high-volume hospitals for both TAVR and SAVR.

## 1. Introduction

Aortic valve replacement is a standard operation for dealing with severe aortic stenosis patients. It was recommended that inoperable or high-risk patients receive transcatheter aortic valve replacement (TAVR), while patients with relatively lower risk can receive surgical aortic valve replacement (SAVR).

The volume-outcome relationship existed in numerous operations. For instance, a group called Leapfrog had recommended certain annual hospital volume thresholds for procedures such as coronary artery bypass surgery (CABG), abdominal aortic aneurysm repair, carotid endarterectomy, and esophagostomy [1]. In the field of percutaneous coronary intervention (PCI) and CABG, the volume-outcome relationship is robust, and there is even a guideline that recommends a specific volume threshold to help reduce mortality [2].

SAVR outcomes were reported to be positively related to hospital volume decades ago. TAVR was brought in 2002 by Cribier et al. [3]; it quickly developed in recent years, and a few studies reported on the volume-outcome relationship of TAVR. However, the volume-outcome relationship in both surgical and transcatheter aortic replacements has not been reviewed. We could recommend an appropriate threshold for aortic replacement procedures if we could identify the volume-outcome relationship. Moreover, we could discuss the reasons why it causes different outcomes among hospitals so that we can provide better health advice. Therefore, we reviewed related studies and performed a meta-analysis to provide a more accurate estimate of the relationship between volume and mortality after TAVR and SAVR.

## 2. Method

### 2.1. Search Strategy and Study Selection

An experienced investigator systemically searched the literature through MEDLINE, EMBASE, PUBMED, and the Cochrane Library up to September 2019. The words “aortic valve replacement,” “aortic stenosis,” “transcatheter aortic valve implantation,” “surgical aortic valve replacement,” “volume,” and “caseload” were used in our searching process. There were no restrictions on language or publication type. Additionally, we searched reference lists of the relevant articles to identify additional items missed before. Two reviewers independently screened titles and abstracts of all reports. Any divergence that appeared was resolved by discussion. As it is not appropriate to mix the odds ratio (OR) for a dichotomous effect estimate with those for a continuous effect estimate, we only included studies using a specific cut-off point to distinguish high-volume from low-volume hospitals. Correction for differences in case mix was required, as these differences may introduce bias. We screened articles using the following inclusion criteria:

1. Specific cut-off points to distinguish high volume and low volume were recorded
2. The volume and short-term mortality (in-hospital or 30-day mortality) relationship was investigated
3. The study contains a representative sample of patients treated at the included center
4. The results should be adjusted for baseline differences in a multivariate model, at least for age and sex
5. The results reported ORs or relative risks (RR), or the odds ratio could be calculated in our consideration (because short-term all-cause mortality in TAVR or SAVR were all less than 10% so RR can be involved)
6. The literature should be full text

### 2.2. Data Extraction and Quality Assessments

Complete articles were retrieved, and the selection criteria was applied by two of the authors, who also extracted the data independently using a standard extraction form. We extracted patients' information with effect estimates and the 95% confidence interval (CI), region, population number, period, database, type of the data, cut-off value, and so on. Two reviewers resolved the disagreements by discussion. If authors reported separate effect estimates for subsequent periods, these were entered individually in the analysis. And quality assessment was done through the Newcastle-Ottawa Scale (NOS) to assess the quality of the observational studies [4]. We carefully examined the data source of every study to prevent including multiple studies, reporting from the same database. If insufficient information were available in the article, we would contact the authors for additional information. All reviewers resolved conflicts through consensus.

### 2.3. Data Interpretation

Our analysis was performed with Stata v13 (StataCorp, College Station, TX). We included only one comparison in our study. That means the highest-volume group is compared with the odds of mortality in the lowest-volume group.

Heterogeneity was assessed through the *Q*-statistic and *I*^2^. Summary estimates were presented as a forest plot, and a fixed (*I*^2^ < 50%) or a random (*I*^2^ > 50%) effects model was performed depending on the *I*^2^ value. A random effects model was assumed to obtain summary estimates. If substantial between-study heterogeneity was observed, subgroup analysis was undertaken to identify the source of heterogeneity. Sensitivity analysis was performed for mortality according to study design. Study bias was examined through plots of standard error by log OR and was tested by Egger's test. The test was set at the 2-sided 0.05 level. The checklist for Meta-analysis Of Observational Studies in Epidemiology (MOOSE) was followed in preparing the manuscript [5].

## 3. Results

**Figure 1:**
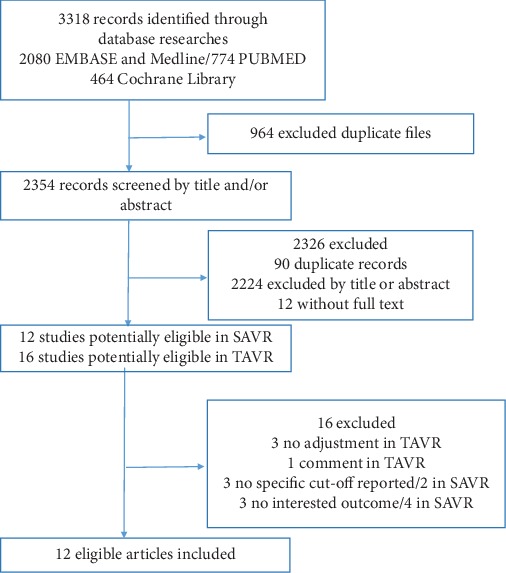
Flowchart of literature review.

In total, we searched 3318 articles (the study selection process is exhibited in Figure 1), and after removing duplicates, 2354 studies were left for primary selection, of which 12 articles were related to the volume-outcome relationship in SAVR and 16 reviews in TAVR. In 12 SAVR articles, six were excluded because no specific cut-off was reported or no interested outcome was recorded. In 16 pieces of literature related to TAVR, ten were excluded because of no particular cut-off, no mortality recorded, and no adjustment of the results or because of article type. All eligible articles were in English. The result of the quality assessment is listed in Supplementary Table 1; all the studies were of high quality. The characteristics of the included articles are shown in Table 1.

### 3.1. Transcatheter Aortic Valve Replacement

Six studies [6–11] comprising 115,596 patients were included and had a population period ranging from 2005 to 2016; three were from the U.S., two were from Germany, and the last one analyzed patients from South and North America and Europe. Two studies used clinical data; the rest used less evidenced administrative data. The reviews got 7-8 scores from the NOS scale (Supplementary Table 1).

**Figure 2:**
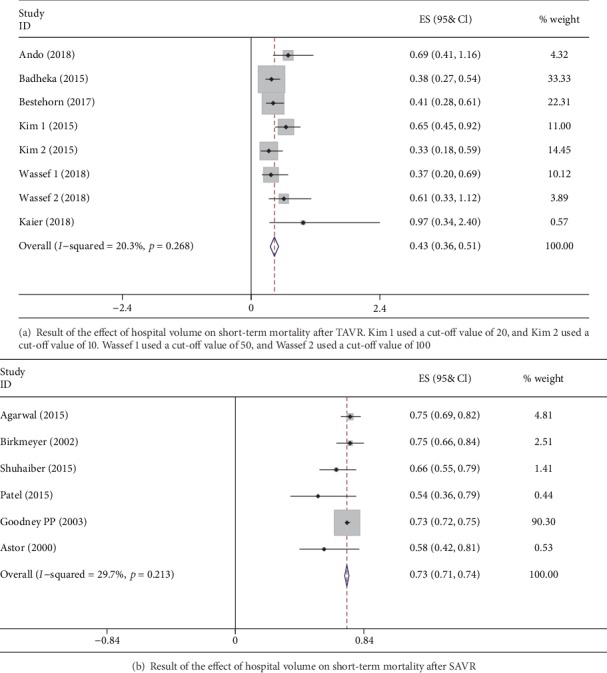
Forest plot of meta-analysis.

**Figure 3:**
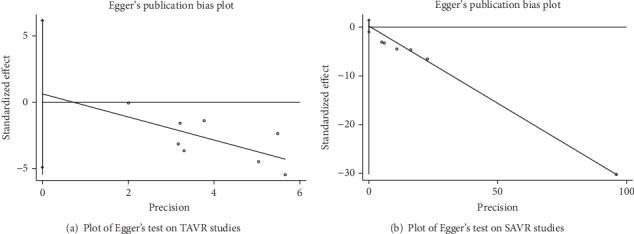
Plots of Egger's test.

The pooled estimated effect was significantly in favor of high-volume hospitals with an OR of 0.43 (CI 0.36-0.51) (Figure 2). Low heterogeneity was observed with an *I*^2^ value of 20.3% (*P* = 0.27). Subgroup analysis was performed by variants of population period, region, data type, and cut-off value. None of these subgroups exhibited a significant change of the pooled estimated effect (Table 2). By omitting Kim et al.'s [10] data using a cut-off value of 10 (in which only transfemoral approach TAVR was included), the heterogeneity reduces to 0 but barely changes the result of 0.41 (CI 0.32-0.49). Egger's test did not exhibit significant publication bias (*P* = 0.79) (Figure 3).

### 3.2. Surgical Aortic Valve Replacement

In total, six studies [12–17] comprising 418,384 patients in more than 1000 hospitals were included. All reviews came from the United States, ranging from 1994 to 2011. The data were all administrative. Data from Goodney et al. [15] was recorded as RR. These studies got 7-8 scores from the NOS scale (Supplementary Table 1).

The meta-analysis revealed that the OR of short-term mortality for a high-volume hospital compared with a low-volume hospital was 0.73 (CI 0.71, 0.74); the forest plot is shown in Figure 2. The *I*^2^ value was 29.7% (*P* = 0.22), demonstrating low-moderate heterogeneity.

Table 2 shows the results of the subgroup analysis of hospital volume and mortality with ORs as effect size. The population period and cut-off value seemed to have no influence on the final results. Further sensitive analysis showed that omitting Patel et al.'s [16] study could reach an *I*^2^ value of 3.2%, but it did not affect the result of 0.73 (CI 0.72-0.74). Eliminating Goodney et al.'s study did not reduce the heterogeneity (*I*^2^ = 41.8%). Egger's regression was not significant to indicate publication bias (*P* = 0.63) (Figure 3).

## 4. Discussion

We systemically searched databases and strictly screened for the literature. As a result, our study indicated an inverse relationship between hospital volume and short-term mortality both in TAVR (0.43, CI 0.36-0.51) and SAVR (0.73, CI 0.71-0.74). Our findings in TAVR were consistent with previous researches. However, de Biasi et al. [18] analyzed 7635 patients receiving TAVR from the 2012 NIS database and found annual hospital TAVR volume as a continuous variable that did not relate to adjusted short-term mortality (1, CI 0.99-1, *P* = 0.11). Because we did not find other researchers who reported volume as a continuous variable, we therefore cannot pool it up. Using institutional capacity as the continuous variable was more convincing than the binary variable, indicating to us that we should analyze this object again in the future with more data.

Apart from the annual hospital volume-mortality relationship, there was a cumulative learning curve that existed in TAVR. Wassef et al. [19] noticed that in-hospital mortality was significantly reduced when it reached a total center procedure volume of 225 cases. Caroll et al. [20] included 42,988 patients from 395 hospitals and found that compared to No. 138-602 cases, the initial cases suffered more mortality (cases 1-30: 1.85, CI 1.55-1.99; cases 31-71: 1.56 CI 1.30-1.86; and cases 72-137: 1.36, CI 1.132-1.639). Arai et al. [21] investigated transaortic TAVR by 2 cardiac doctors and revealed that the occurrence of adverse events such as life-threatening bleeding, stroke, and acute kidney injury was significantly decreased after the initial 128 cases for each operator (9% vs. 1%, *P* = 0.002; 5% vs. 0%, *P* = 0.014; and 16% vs. 6%, *P* = 0.002, respectively). However, there is no operator volume-outcome relationship in TAVR reported yet. Previous PCI investigations might draw a rough outline for TAVR. Strom et al. [22] included 23 studies of high quality and performed a meta-analysis revealing that mortality and major adverse cardiac events increase as operator volumes decrease in PCI.

We also found that there was an inverse relationship between yearly hospital volume and short-term mortality in SAVR. Gonzalez et al. [23] investigated 23,353 patients from 2005 to 2006 through the Medicare Provider Analysis and Review database, and they found that compared to the highest-volume hospital, the patients from the lowest-volume hospital were more likely to die (1.63, CI 1.47-1.81), develop major complications (1.12, CI 1.06-1.18), and experience failure-to-rescue events (1.57, CI 1.38-1.79). Reames et al. [24] categorized volume quintiles based on increasing two-year hospital volume and involved 292,901 patients from 2000 to 2009; their results show that high-volume hospitals had lower mortality than the low groups each year. Idrees et al. [25] identified 360,437 patients undergoing isolated surgical AVR between 1998 and 2011 from the NIH database, and they concluded that high-volume centers had lower odds of stroke in medium-risk and high-risk patients (0.59, CI 0.37-0.94 and 0.39, CI 0.22-0.68, respectively).

We found that the relationship between hospital volume and mortality was widely reported in different surgeries. Halm et al. [26] reviewed 135 articles covering 27 procedures and noticed that higher capacity was associated with better outcomes. Furthermore, from among 27 procedures, pancreatic cancer, esophageal cancer, abdominal aortic aneurysms, and pediatric cardiac surgeries exhibited the most robust relation.

Based on the above information, we quickly found that the volume-outcome relationship widely existed. Some reasons may explain it. First was that effective treatments were more often used by high-volume hospitals or physicians than their low-volume counterparts. Secondly, operators from the high-volume centers would accumulate more experience than those of the low-volume centers as caseload and time increased. Maruthappu et al. [27] had proven that experience was vital in operative procedures. They reviewed 57 studies of 35 procedure types and quantified experience as caseload, annual case volume, and years of practice; finally, they concluded that increasing expertise was associated with improved performance. Third, innovations like significant trials and new devices were likely implemented in the high-volume centers, which were always accompanied with an improvement of health care. For example, Columbia University Medical Center, as a high-volume center, was involved in PARTNER I and PARTNER II trials aimed at TAVR indication expansion, CLEAN-TAVI trial aimed at cerebral protection device employment, and SAPIEN 3 valve registry aimed at exploring the safety of new generation devices [28].

We can conclude that higher volume hospitals were associated with better short-term outcomes. When it comes to clinical application, there were still some points brought to our attention. First, we cannot tell the specific cut-off value of TAVR and SAVR to distinguish high and low volume because no significance was found between different groups. So, did this situation happen to other meta-analysis studies about the volume-outcome relationship [29, 30]? Second, if the volume-outcome relationship was investigated year by year, we may notice a magnitude attenuation in more recent years [9]. It can be explained by the plateau of the longitudinal learning curve [27]. Operators from high-volume hospitals accumulate experience rapidly earlier in time and soon reach a plateau. On the other hand, operators from low-volume hospitals slowly accumulate experience. At last, after the plateau appears in high-volume hospitals, the gap between two types of hospitals gradually diminished. Third, TAVR was widely implemented, and indications of TAVR were expanded. It is hard to ignore the interactive effect between TAVR and SAVR. Mao et al. [31] found that high SAVR volume hospitals were likely to develop a TAVR procedure fast, and high TAVR volumes had lower mortality after the procedure, particularly when hospitals have high SAVR volumes [31]. As TAVR indication now shifts to lower-risk patients, the patients' proportion in TAVR and SAVR may change so that we can expect the decrease of mortality with better patient selection.

Our study was the first meta-analysis focused on the hospital-outcome relationship in severe aortic stenosis receiving TAVR or SAVR. We systemically reviewed related articles and selected publications with high quality to include in our study. One limitation of our study was that the population period of SAVR was approximately 9-24 years ago, which impaired its value if applied to a new era. Secondly, the majority of data came from administrative resources, which were less evident than clinical data.

## 5. Conclusion

In short, high-volume hospitals can reduce the short-time mortality of patients receiving SAVR or TAVR compared to the lower in-hospital mortality of those treated at low-volume hospitals.

## Figures and Tables

**Table 1:** Characteristics of included studies.

Name	Year	Operation	Lowest cut-off	Highest cut-off	Population	Hospital	Region	Period	Source/database	Data type
Agarwal	2015	SAVR	50	100	104,699	Approximately 1000	USA	2002-2011	Nationwide inpatient sample	Administrative
Birkmeyer	2002	SAVR	200	550	151,610	1069	USA	1994-1999	Centers for Medicare and Medicaid services	Administrative
Shuhaiber	2015	SAVR	8	19	87,675	Approximately 1000	USA	1998-2011	Nationwide inpatient sample	Administrative
Patel	2015	SAVR	98	98	6270	33	USA	2008-2011	Centers for Medicare and Medicaid services	Administrative
Goodney PP	2003	SAVR	230	230	59,389	Na	USA	1994-1999	The national Medicare database	Administrative
Astor	2000	SAVR	60	180	8741	176	USA	1994	Nationwide inpatient sample	Administrative
Ando	2018	TAVR	30	130	48,886	Na	USA	2011-2015	Nationwide inpatient sample	Administrative
Badheka	2015	TAVR	5	20	1481	Na	USA	2012	Nationwide inpatient sample	Administrative
Bestehorn	2017	TAVR	50	200	9924	87	Germany	2014	German Quality Assurance Registry on Aortic Valve Replacement of the Federal Joint Committee	Clinical
Kim	2015	TAVR	20 of TF 10 of TA	20 of TF 10 of TA	7660	256	USA	2012	Nationwide inpatient sample	Administrative
Wassef	2018	TAVR	50 and 100	50 and 100	3468	16	America and Europe	2005-2016	The international TAVR registry	Clinical
Kaier	2018	TAVR	50	100	43,996	113	Germany	2008-2014	Diagnosis-related group statistics	Administrative

**Table 2:** Results of sensitivity and subgroup analyses.

Procedure	Subgroup	Odd ratio (95% CI)	*P* value
TAVR	Population period		0.61
	Data before 2010 included	0.43 (0.34, 0.51)	
	Data before 2010 excluded	0.46 (0.25, 0.66)	
TAVR	Region		0.93
	USA	0.44 (0.34, 0.53)	
	Europe	0.42 (0.26, 0.59)	
	North and South America and Europe	0.44 (0.23, 0.64)	
TAVR	Data type		0.53
	Administrative	0.44 (0.34, 0.54)	
	Clinical	0.42 (0.29, 0.55)	
TAVR	Cut-off for lowest volume		0.93
	<50	0.44 (0.34, 0.53)	
	≥50	0.43 (0.3, 0.56)	
	Cut-off for highest volume		0.89
	<100	0.41 (0.32, 0.51)	
	≥100	0.48 (0.34, 0.62)	
TAVR	Outcome		0.78
	In-hospital mortality	0.43 (0.35,0.52)	
	30-day mortality	0.44 (0.23,0.64)	
SAVR	Population period		0.84
	Data before 2000 included	0.73 (0.71, 0.74)	
	Data before 2010 excluded	0.72 (0.66, 0.77)	
SAVR	Cut-off for lowest volume		0.6
	<100	0.71 (0.65, 0.76)	
	≥100	0.73 (0.72, 0.75)	
	Cut-off for highest volume		0.1
	<100	0.63 (0.53, 0.74)	
	≥100	0.73 (0.72, 0.75)	

## Abbreviations

TAVR: Transcatheter aortic valve replacement
SAVR: Surgical aortic valve replacement
PCI: Percutaneous coronary intervention
CABG: Coronary artery bypass surgery
CI: Confidence interval
OR: Odds ratio
RR: Relative risk
CLEAN-TAVI trial: CLaret Embolic Protection ANd TAVI trial
PARTNER trial: Placement of AoRTic TraNscathetER Valve trial.
NOS: Newcastle-Ottawa Scale.

## Supplementary Materials

Supplementary Materials Results of literature quality assessment.
