# Supplementary material for: The Relation between Volume and Outcome of Transcatheter and Surgical Aortic Valve Replacement: A Systematic Review and Meta-Analysis
Source: Cardiovasc Ther. 2020 Apr 18;2020:2601340. doi: 10.1155/2020/2601340 (PMC7189304; doi:10.1155/2020/2601340)
Supplement: Supplementary Materials — Results of literature quality assessment. [file 2601340.f1.docx]

Supplementary Table 1：Results of literature quality assessment


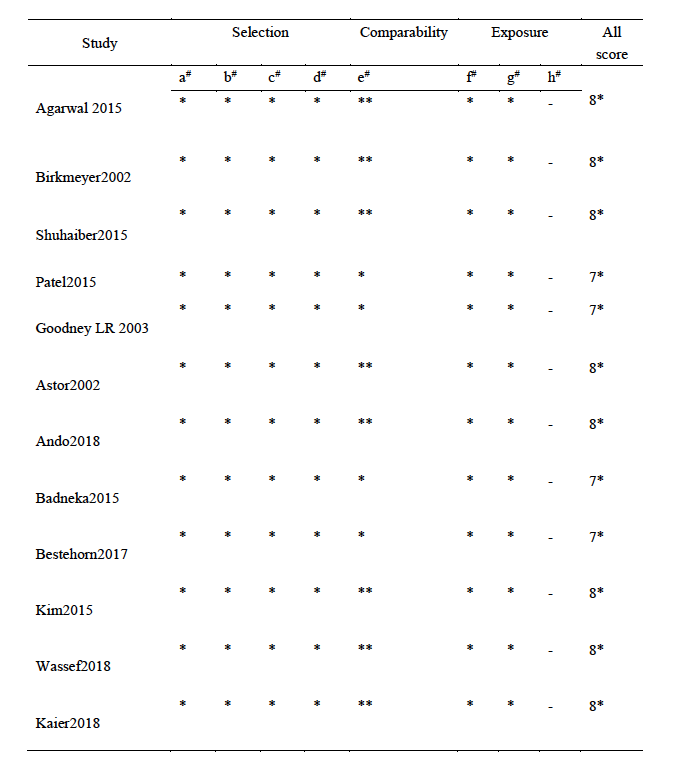


*：stand for the study can be awarded one star in corresponding question; a^#^: case definition; b^#^:Representative of the case; c^#^:Selection of control; d^#^:Definition of control; e^#^:Study control age and sex to reduce bias; Study control other complication as additional factors; f^#^:Ascertainment of exposure; g^#^:Same method of ascertainment for cases and control; h^#^:Non-respond rate
